# Cathepsin X Cleaves Profilin 1 C-Terminal Tyr139 and Influences Clathrin-Mediated Endocytosis

**DOI:** 10.1371/journal.pone.0137217

**Published:** 2015-09-01

**Authors:** Urša Pečar Fonović, Janko Kos

**Affiliations:** 1 Faculty of Pharmacy, University of Ljubljana, Ljubljana, Slovenia; 2 Department of Biotechnology, Jožef Stefan Institute, Ljubljana, Slovenia; Thomas Jefferson University, UNITED STATES

## Abstract

Cathepsin X, a cysteine carboxypeptidase, is upregulated in several types of cancer. Its molecular target in tumor cells is profilin 1, a known tumor suppressor and regulator of actin cytoskeleton dynamics. Cathepsin X cleaves off the C-terminal Tyr139 of profilin 1, affecting binding of poly-L-proline ligands and, consequently, tumor cell migration and invasion. Profilin 1 with mutations at the C-terminus, transiently expressed in prostate cancer cells PC-3, showed that Tyr139 is important for proper function of profilin 1 as a tumor suppressor. Cleaving off Tyr139 prevents the binding of clathrin, a poly-L-proline ligand involved in endocytosis. More profilin 1—clathrin complexes were present in PC-3 cells when cathepsin X was inhibited by its specific inhibitor AMS36 or silenced by siRNA. As a consequence, the endocytosis of FITC-labeled dextran and transferrin conjugate was significantly increased. These results constitute the first report of the regulation of clathrin-mediated endocytosis in tumor cells through proteolytic processing of profilin 1.

## Introduction

Cathepsin X, a cysteine cathepsin with carboxymonopeptidase activity [1], is greatly upregulated in certain types of cancer, including prostate cancer [2,3]. Cathepsin X influences the motility and adherence of tumor cells and thus, most probably, participates in the epithelial to mesenchymal transition [3]. Adhesive and migrative properties of tumor cells can be altered through either binding to or proteolytic cleavage of integrin receptors [4,5] or by bypassing cell senescence [6]. As shown on a PymT-induced mouse model of breast cancer, cathepsin X promotes early tumor formation, tumor progression and metastasis in the lung, exhibiting functional redundancy with the related cysteine cathepsin B [7]. Profilin 1 has recently been identified as a target for cathepsin X carboxypeptidase activity in tumor cells [8]. The C-terminal part of profilin 1 constitutes a binding site for a plethora of ligands, including phosphatidylinositol lipids and poly-L-proline ligands involved in membrane trafficking, focal contacts, and Rac/rho and cdc42 signalling (reviewed by Witke [9]). The poly-L-proline binding site includes Trp3 and Tyr6 on the N-terminal helix, His133, Leu134 and Tyr139 on the C-terminal helix and Trp31 on strand 2, which is close to Trp3 [10,11]. Mutants of profilin 1 have been prepared over the last 20 years [12,13] in attempts to determine the binding sites for different ligands, however it is still not known how the specificity and affinity of the binding are enabled. Nevertheless, the phosphorylation of either Ser137 or Tyr139 in profilin 1 has been shown, in EGF-stimulated cells, to abolish the binding of poly-L-proline [11,14].

Clathrin, a protein involved in the formation of endocytic coated vesicles, has been recognized as one of the profilin1 poly-L-proline ligands [15]. In contrast to other poly-L-proline ligands, like vasodilator-stimulated phosphoprotein (VASP) and the mammalian homolog of *Drosophila* enabled (Mena), that utilize continuous stretches of eight or more prolines for binding, clathrin exhibits two or more ZPPX motifs [15].

As shown earlier, inhibition of cathepsin X increases the affinity of clathrin binding for profilin 1 [8]. The aim here was to mutate residues at the C-terminus of profilin 1 to assess their contribution to profilin 1 processing, clathrin binding, clathrin-mediated endocytosis and tumor cell migration and invasion. We demonstrated that mutant lacking C-terminal tyrosine decreased endocytosis and actin polymerization and increased cell migration and invasion.

## Materials and Methods

### Preparation of profilin 1 mutants

DNA sequences for mutated forms of profilin 1 were designed and DNA synthesis ordered from GeneArt AG (Life Technologies). *E*. *coli* TOP 10 bacterial cells (Invitrogen) were transformed with plasmids incorporating mutated profilin 1 and carrying ampicillin resistance. Mutated profilin 1 nucleotide sequences were excised with EcoRI (New England Biolabs) and Bam HI (New England Biolabs), ligated into pcDNA3 vector (Invitrogen) and transformed in *E*.*coli* TOP10 cells. Sequences were verified at GATC Biotech.

### Cell culture and transfection

Human prostate cancer cells PC-3 (obtained directly from ATCC, Manassas, USA; ATCC Number: CRL-1435) were cultured in Advanced DMEM (Gibco) and F-12 (1:1) with 10% FBS, 1% L-glutamine and 1% penicillin/streptomycin. Cathepsin X was transiently silenced using Lipofectamine 2000 (Invitrogen) and cathepsin X specific small interfering RNA (Santa Cruz) according to the Instruction Manual. Also, using Lipofectamine 2000, PC-3 cells were transfected with prepared plasmids and profilin 1 mutants were transiently expressed. Tests were performed 48 hours after transfection.

Cathepsin X was inhibited with its specific irreversible inhibitor AMS36 [16,17] at 10 μM concentration for 24 hours. 0.1% dimethylsulphoxide (DMSO) was used as a control.

### Preparation of cell lysates

Cell lysates were prepared 24, 48 and 72 hours after plasmid transfection in lysis buffer (50 mM HEPES pH 6.5, 1 mM EDTA, 150 mM NaCl, 1% Triton X-100) containing protease inhibitor cocktail (Thermo Scientific). For assessment of cathepsin X silencing lysis buffer, containing 50 mM Na acetate pH 5.5, 1 mM EDTA 100 mM NaCl, 0.25% Triton X-100, was used. Total protein concentration was determined by DC Protein Assay (Bio Rad) according to instructions.

### Western blot

Proteins were separated on 12% Tris-glycine gels and transferred to a Hybond-N nitrocellulose membrane (GE Healthcare). The membranes were blocked with 5% skimmed milk powder in phosphate buffered saline (PBS) for 1 hour, then incubated, first with primary antibodies in PBS with 0.05% Tween (PBST) for 1 hour and then with secondary antibodies in PBST for 45 min. Proteins were detected with a SuperSignal West Dura Extended Duration Substrate chemiluminescence kit (Thermo Scientific). FLAG-tagged proteins were detected by blotting according to the instructions for anti-FLAG M2 antibody (Sigma-Aldrich). Proteins were detected with SuperSignal West Femto chemiluminescence kit (Thermo Scientific). Cathepsin X was detected by blotting according to the instructions (R&D Systems). Information regarding antibodies is presented in S1 Table.

### Quantitative ELISA

Quantitative ELISA was done as reported [8].

### Real time cell migration and invasion assays

Cell assays were performed on a Real-Time Cell Analyzer Dual Plate (RTCA DP) Instrument, xCELLigence System (ACEA Biosciences) [18]. The method is based on real-time monitoring of cell invasion, migration or adhesion and captures cell responses during the entire course of the experiment. In the migration assay, CIM plates (cell invasion and migration) were coated with fibronectin (10 μg/ml; BD Biosciences) on the down-side of the microporous PET membrane for 30 min at room temperature and on the upper-side for 2 hrs at 37°C. Excess fibronectin was removed and wells were washed with phosphate buffer saline (PBS). Lower chambers were filled with complete medium and upper chambers with serum-free medium. 2×10^4^ cells were plated per well. Cathepsin X specific inhibitor AMS36 at 10 μM concentration was added as required to the upper and lower chambers. DMSO (0.1%) was used as a control. When using cathepsin X [19], 2 μM enzyme was added to the upper and lower chambers.

In the invasion assay, the down-side only of the membrane was coated with fibronectin and the upper-side with 50% Matrigel (20 μl; BD Biosciences) in serum-free medium (30 min at 37°C). 3×10^4^ cells were plated per well.

### Endocytosis of FITC-dextran and transferrin Alexa Fluor 555 conjugate

For endocytosis studies, 10^4^ cells/well were plated on a 12-well plate. When 70–80% confluence was reached, FITC-labeled dextran (10 kDa; 300 μg/ml; Life Technologies) was added and the cells incubated at 37°C for 2 hours. The plates were then placed on ice, trypsinized and washed twice with PBS. Internalization of transferrin was performed as described previously [20] with modifications: cells were serum starved in internalization medium (Advanced DMEM and F-12 (1:1) with 10 mM HEPES, 0.5% BSA, pH 7.4) for 30 minutes at 37°C, trypsinized and incubated with transferrin Alexa Fluor 555 conjugate (Molecular Probes; 50 μg/ml) for different time periods at 37°C. Internalization was stopped by putting cells on ice. Cells were then washed with acid (50 mM glycine, 150 mM NaCl, pH 3) and neutralized (PBS, 0.5% BSA) to remove transferrin, bound to the cell surface and non-bound transferrin. To confirm that internalization of transferrin Alexa Fluor is receptor-mediated, experiment was repeated in the presence of 100-fold excess of non-labeled holo-transferrin (Sigma-Aldrich).

Both fluorescences were measured by FACSCalibur flow cytometer using Cell Quest software and the data analyzed with FlowJo software.

### Cytotoxicity assay

The optimal concentration of chlorpromazine (Sigma), the inhibitor of clathrin-mediated endocytosis, was determined by plating 10^4^ cells/well on a 96-well plate for 24 hours. They were incubated with various concentrations of chlorpromazine (30 min, 37μC) prior to the addition of MTS reagent (CellTiter 96 Aqueous One Solution Cell Proliferation Assay, Promega). A_492_ was measured after 80 minutes.

### Actin polymerization

10^4^ cells/well were plated on a 12-well plate. When 70% to 80% confluence was reached they were transfected with plasmids carrying mutants of profilin 1. After 48 hours the cells were trypsinized, fixed with 10% formalin for 10 min on ice, and then permeabilized with 0.5% Tween 20 in PBS for 15 min at room temperature in the dark. Cells were resuspended in PBS and incubated with phalloidin-tetramethylrhodamine B isothiocyanate conjugate (Sigma) for 1h on ice in the dark. After washing twice with PBS, the fluorescence signal was analyzed on a FACSCalibur flow cytometer with Cell Quest software and the data analyzed with FlowJo software.

### Fluorescence microscopy analysis

Coverslips were coated overnight at 4°C with fibronectin (10 μg/ml). 3×10^3^ cells/well were plated and AMS36 or DMSO added at the required confluence. After 24 hours FITC-labeled dextran (150 μg/μl) was added for 2 hours at 37°C and the plates then placed on ice, washed three times with ice-cold medium, fixed with 500 μl formaldehyde for 1 hour and washed once with PBS. Prolong Antifade kit (Molecular Probes) was used for mounting the coverslips on glass slides. A Zeiss LSM 710 confocal microscope with ZEN 2012 image software was used for fluorescence microscopy.

### Proximity ligation assay (PLA)

PLA (Duolink In Situ–Sigma Aldrich) was carried out according to the Instruction Manual. 8×10^3^ cells/well were plated on fibronectin (10 μg/ml) coated coverslips. AMS36 (10 μM) or DMSO were added 24 hours prior to the assay. Cells were fixed in 10% formalin (Sigma-Aldrich) for 45 min, permeabilized in 0.5% Tween 20 in PBS for 10 min, blocked in 3% bovine serum albumin (BSA) in PBS for 30 min then incubated with rabbit anti-profilin antibody (S2 Table) and mouse anti-clathrin antibody (S2 Table) in 3% BSA in PBS for 2 hours at room temperature. Cells were further assayed using PLA probe anti-rabbit PLUS and PLA probe anti-mouse MINUS, following the Instruction Manual in detail. A Zeiss LSM 710 with ZEN 2012 image software was used for confocal microscopy.

## Results

### Expression of profilin 1 mutants in PC-3 cells

**Fig 1:**
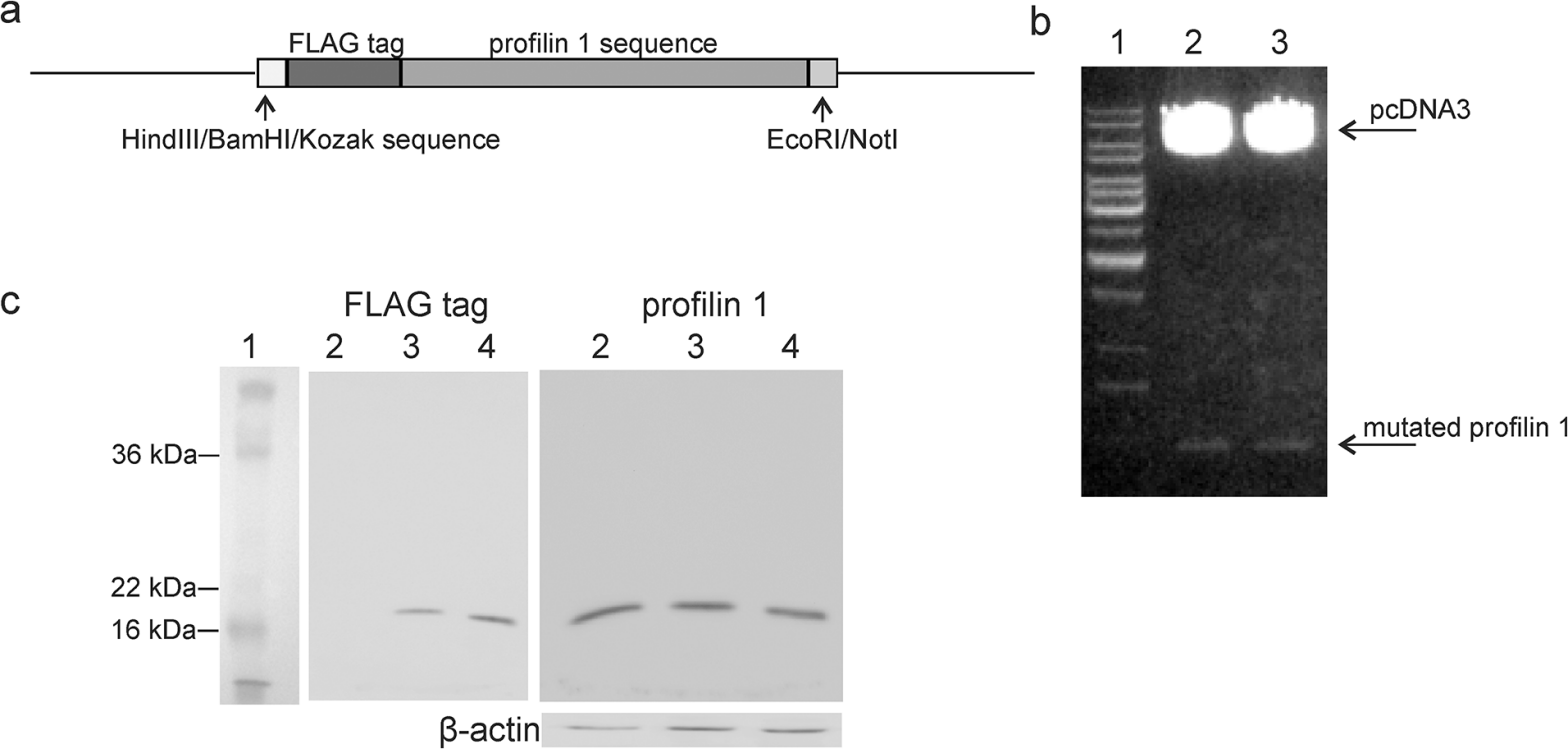
Expression of profilin 1 mutants in PC-3 cells. (A) Scheme of the DNA construct of profilin 1 mutants. (B) Restriction analysis of the DNA construct ligated into the pcDNA3 cloning vector using EcoRI and BamHI. Line 1: GeneRuler 1 kb DNA Ladder (Promega), line 2: restriction of Pfn1-Tyr139 mutant, line 3: restriction of Pfn1-Q138P mutant. (C) Western blot analysis of transfected PC-3 cell lysates expressing different profilin 1 mutants. Cell lysates were prepared 48 h post transfection. Line 1: size marker (SeeBlue® Pre-stained Protein Standard, Life Technologies), line 2: transfected empty pcDNA3 vector, line 3: transfected Pfn1-Tyr139/pcDNA3, line 4: transfected Pfn1-Q138P/pcDNA3. Mutants were detected with anti-FLAG antibodies. Detection of profilin 1 (native and mutated) and β-actin on the same membrane is also shown. Due to the chemiluminescent detection, size marker is added as a separate strip.

Two mutants of human profilin 1 were prepared, based on the result that cathepsin X cleaves the terminal Tyr139 from recombinant profilin 1 [8]. Mutant 1 lacks the terminal Tyr (Pfn-Tyr139). Mutant 2, with Gln138 changed to Pro138 (Pfn-Q138P), should prevent C-terminal cleavage of profilin 1 since cathepsin X does not favor Pro at the P2 position. The DNA constructs (Fig 1A) were synthesized, cloned into mammalian pcDNA3 vector (Fig 1B) and, after sequence verification, used to transfect prostate cancer PC-3 cells. Expression of each profilin 1 mutant was confirmed after 48 hours with Western blot (Fig 1C). As a control, PC-3 cells were transfected with empty pcDNA3 vector. Total profilin 1 (native and mutated) was detected with anti-profilin 1 antibody.

### Pfn-Tyr139 increases migration and invasion of PC-3 cells

**Fig 2:**
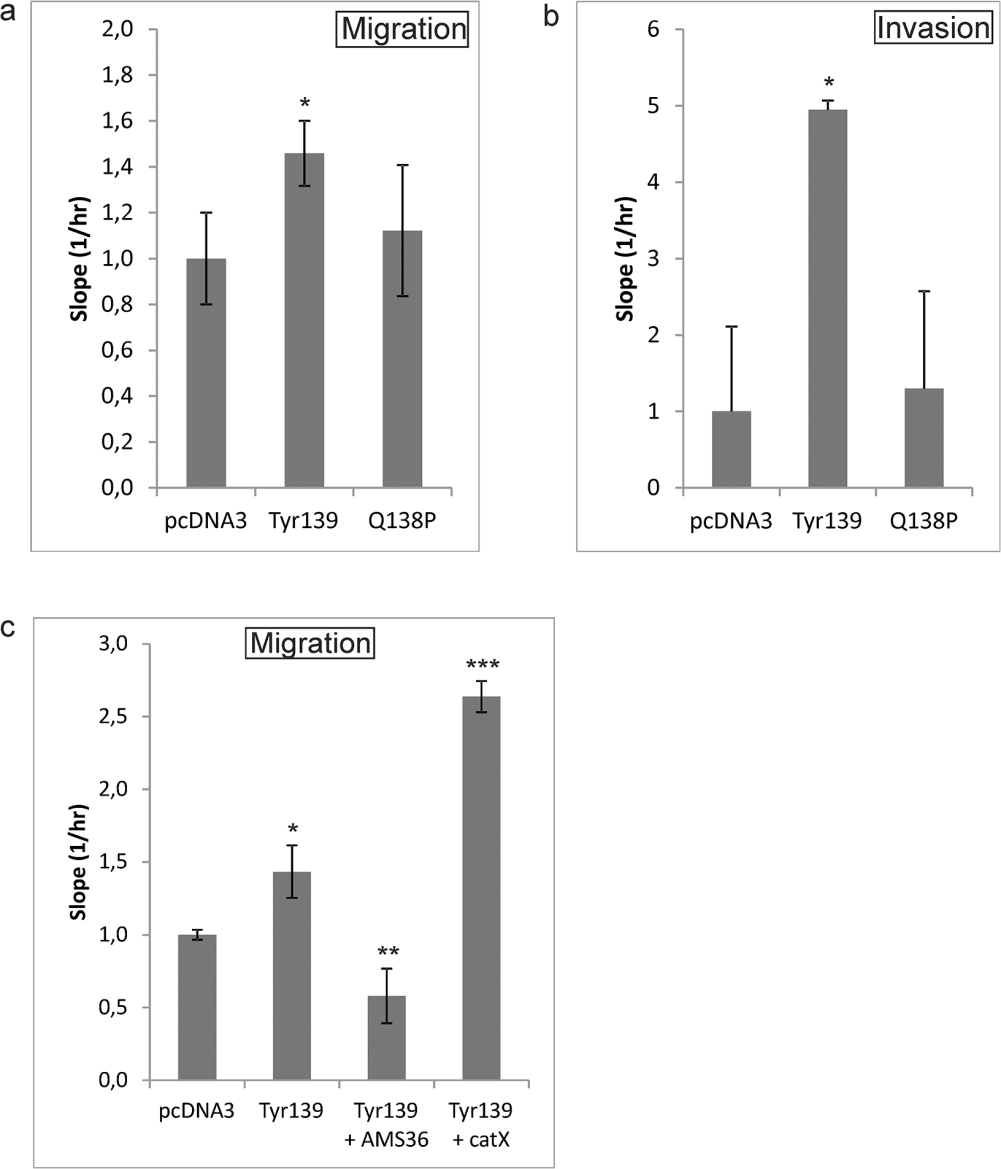
Migration and invasion of PC-3 cells transfected with profilin1 mutants. The level of cell migration/invasion, assessed by increases in the curve slopes (1/h), is shown. All results were normalized to cells transfected with empty pcDNA3 vector (the control). Assays were carried out in triplicate. (A) Results from migration were calculated for the interval between the 45 hour and 98 hour time points, during which the slopes of the curves were linear. (B) Results from invasion were calculated for the interval between the 49 hour and 107 hour time points. (C) The influence of AMS36 inhibitor (10 μM) and recombinant cathepsin X (2 μM) was tested on the cells transfected with Pfn1-Tyr139 mutant. Results of migration were calculated for the interval between the 29 hour and 38 hour time points. *P≤0.05 **P<0.01; ***P<0.001.

Migration and invasion were assayed 26 hours post transfection of PC-3 cells and then every 15 minutes for 3 days. In both assays only cells transfected with Pfn1-Tyr139 showed significantly increased migration and invasion (Fig 2A and 2B). Migration was also assayed in the presence of AMS36 (10 μM) or of recombinant cathepsin X (2 μM). As expected, AMS36 inhibited cathepsin X action on native profilin 1 and decreased cell migration, while the recombinant enzyme produced more truncated profilin 1 molecules and thus increased the migration (Fig 2C).

### Intact, but not cleaved, profilin 1 forms complexes with clathrin

**Fig 3:**
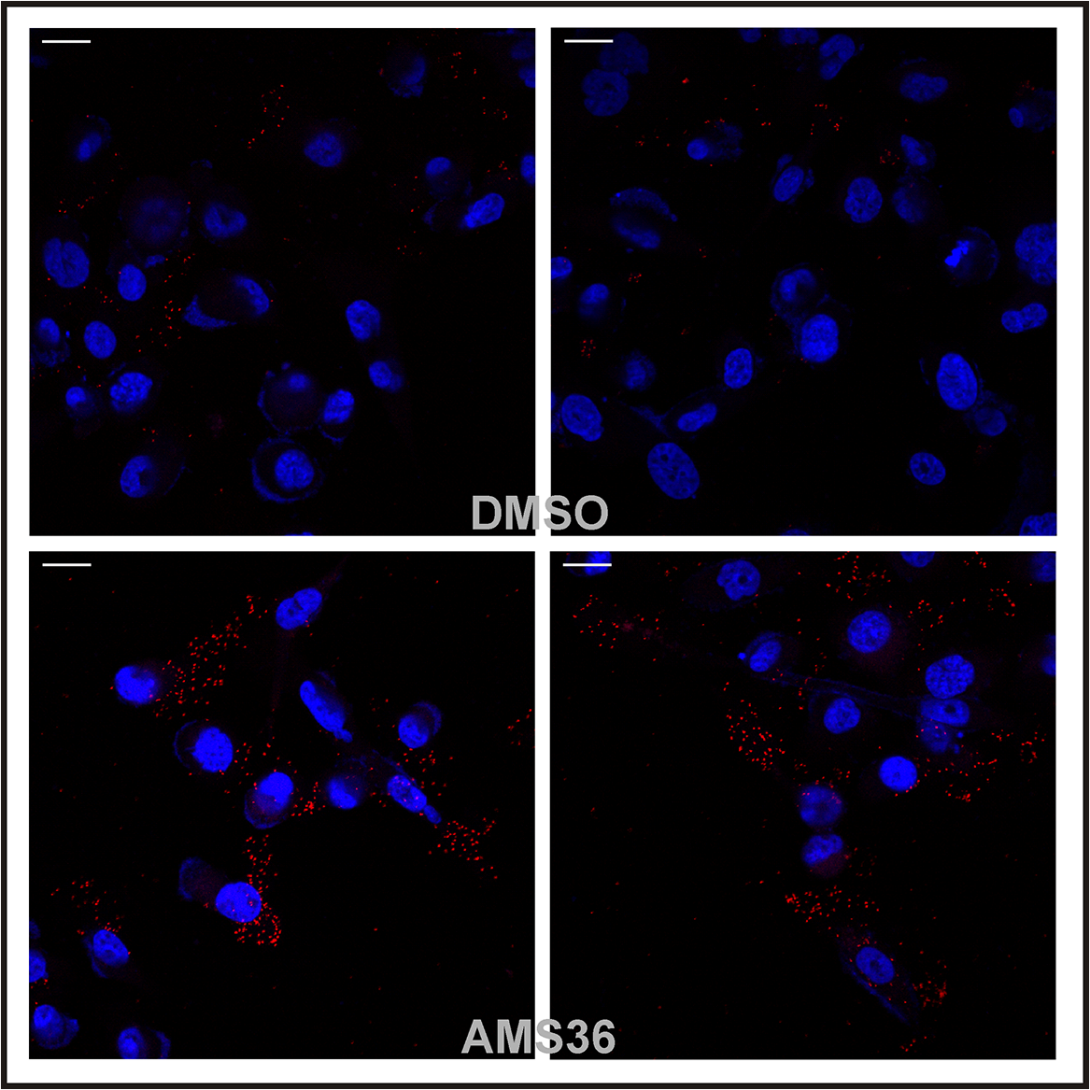
Presence of the profilin 1—clathrin complex is cathepsin X dependent. Cells were treated with cathepsin X inhibitor AMS36 (10 μM) or DMSO. Profilin 1—clathrin complexes were detected with a proximity ligation assay and analyzed with confocal microscopy. Each red dot represents a Texas red signal present on the spot with the complex. Cell nuclei were stained with DAPI. Bar, 20 μm.

The association of profilin 1 with clathrin is cathepsin X dependent. As shown in our previous study on cathepsin X knock-down PC-3 cells [8], 80% more clathrin was co-immunoprecipitated with profilin 1 than in wild type cells. The two proteins co-localized in regions close to the membrane. The stability of the profilin 1∼clathrin complex was further confirmed to depend on cathepsin X activity by using a proximity ligation assay, which detects close association between two proteins [21] (Fig 3). The red signals on Fig 3 show the close proximity of profilin1 and clathrin, typical of protein complexes. In control cells the signals are scattered and few, whereas in cells treated with cathepsin X inhibitor AMS36 they are much more extensive, revealing that intact rather than cleaved profilin 1 binds clathrin.

### Endocytosis of FITC-labeled dextran and transferrin Alexa Fluor 555 conjugate is cathepsin X dependent

**Fig 4:**
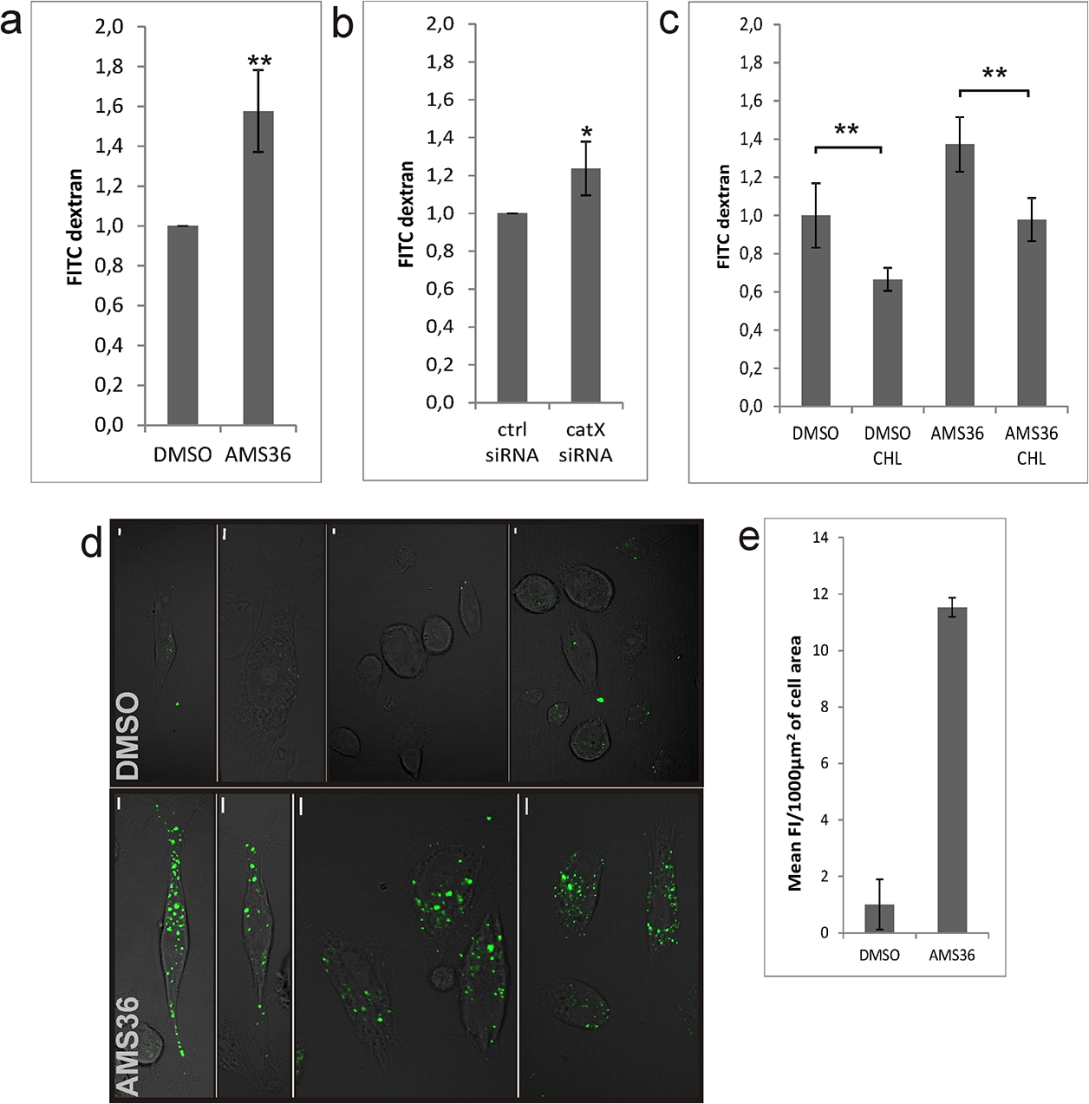
Endocytosis of fluorescein-labeled dextran is abrogated by cathepsin X. Clathrin-mediated endocytosis of fluorescein-labeled dextran (10 kDa) was followed by flow cytometry. Cathepsin X in cells was (A) treated with cathepsin X inhibitor AMS36 (10 μM) or (B) silenced with its specific siRNA. Control cells were treated with DMSO or transfected with control siRNA. (C) Prior to the inhibitor treatment, cells were treated with 10 μM chlorpromazine (CHL). Median values of two or three independent experiments (each in triplicate) are shown. *P<0.05; **P<0.01 (D) Endocytosis of fluorescein-labeled dextran was followed under the microscope. Representative examples of DMSO and AMS36 treated cells are shown. (E) Areas and mean intensity values of the cells were measured using ZEN 2012 software and the mean intensity/1000 μm^2^ of the cell area calculated. 28 (DMSO) and 18 (AMS36) cells were measured. Bars, 5 μm.

Clathrin-mediated endocytosis was studied using FITC-labeled dextran (10 kDa) and transferrin Alexa Fluor 555 conjugate. Treatment with AMS36, as shown by flow cytometry, increased endocytosis of FITC-labeled dextran by 50% with respect to the control (Fig 4A). Similarly, endocytosis increased by 25% in cathepsin X knock-down cells (Fig 4B). The extent of cathepsin X depletion is shown in S1 Fig. The use of chlorpromazine, an inhibitor of clathrin-mediated endocytosis, neutralized the effect of AMS36 (Fig 4C). Since chlorpromazine can be strongly cytotoxic, its cytotoxicity toward PC-3 cells was assayed. It was not cytotoxic at 10 μM concentration (S2 Fig) and this concentration was therefore used in all further experiments. Increased uptake of FITC-dextran was also demonstrated with microscopy, where the average fluorescence intensity of DMSO and AMS36 treated cells per 1000 μm^2^ was 0.25 and 2.85, respectively (Fig 4D and 4E).

**Fig 5:**
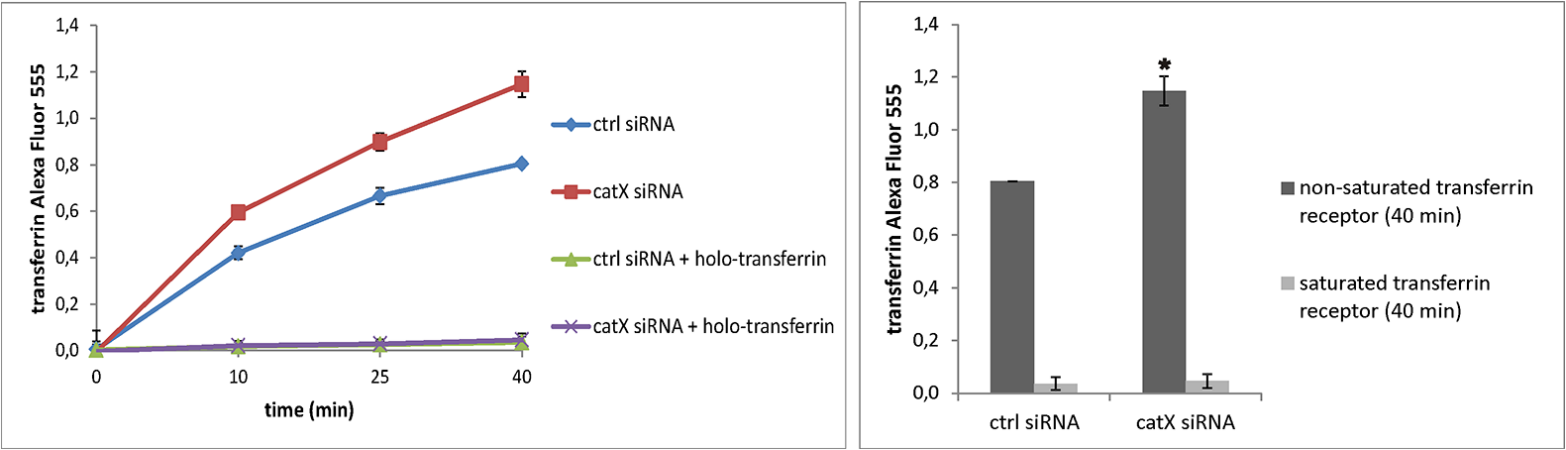
Endocytosis of transferrin Alexa Fluor 555 conjugate is abrogated by cathepsin X. Clathrin-mediated endocytosis of transferrin Alexa Fluor 555 conjugate was followed by flow cytometry. Cathepsin X in cells was silenced with its specific siRNA, control cells were transfected with control siRNA. Mean values of three independent experiments (each in duplicate) are shown. In a control experiment transferrin receptor was saturated with holo-transferrin. *P<0.05.

Internalization of transferrin Alexa Fluor 555 conjugate was studied at four time points (Fig 5). Endocytosis in cathepsin X knock-down cells increased by 30% after 40 minute incubation at 37°C. With saturated transferrin receptor there was no clathrin-mediated endocytosis (Fig 5).

### Pfn1-Tyr139 affects endocytosis of FITC dextran

**Fig 6:**
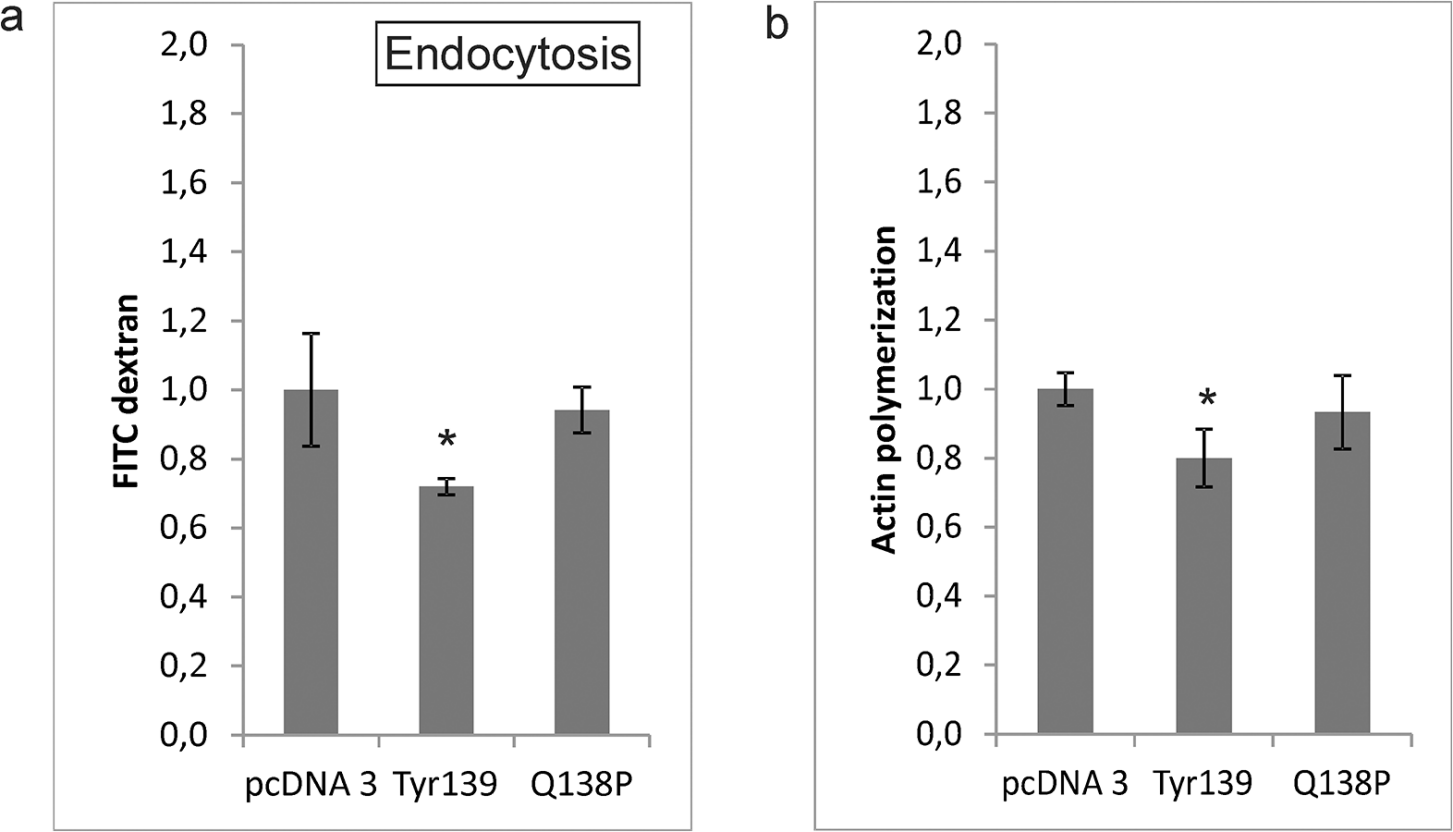
Profilin 1 mutant Pfn1-Tyr139 decreases endocytosis and actin polymerization. (A) Cells were transfected with two plasmids carrying different profilin 1 mutants and empty plasmid (control). Clathrin-mediated endocytosis of fluorescein-labeled dextran (10 kDa) was followed with flow cytometry. Median values are representative of two independent experiments (one in triplicate and one in duplicate). *P<0.05 (B) Cells were transfected with different profilin 1 mutant constructs. After 48 hours filamentous actin was stained with phalloidin conjugate and analyzed with flow cytometry. Mean values are representative of two independent experiments (one in triplicate and one in duplicate). *P<0.05.

The effects of the two profilin 1 mutants on endocytosis of FITC dextran 48 hours post transfection were determined. Only Pfn1-Tyr139 decreased endocytosis–the difference in endocytosis caused by Pfn1-Q138P was not statistically significant (Fig 6A).

### Pfn-Tyr139 decreases the polymerization of actin

Both profilin 1 mutants were assayed for their effects on actin polymerization 48 hours post transfection. Again, Pfn1-Tyr139 decreased polymerization while the difference in actin polymerization caused by Pfn1-Q138P was not statistically significant (Fig 6B).

## Discussion

Profilin 1 can bind a large variety of ligands and, by that, influences important cell processes. In cancer its tumor suppressive functions are impaired, possibly due to changes in ligand binding with resulting loss of control in actin polymerization, cell migration and endocytosis. The binding of ligands to profilin 1 depends on the structure of the C-terminal part and our study demonstrates that cleavage of the C-terminal tyrosine by cathepsin X drastically changes the ligand binding and function of profilin 1.

The binding of ligands to profilin 1 is regulated by various mechanisms. Among these is phosphorylation of either Tyr139 or Ser137, which prevents binding of poly L-proline [11,22]. VEGF-A-inducible phosphorylation of Tyr129 has been reported to be critical for the migration and angiogenesis of endothelial cells [23]. Phosphorylation of Tyr129 is also associated with tumor angiogenesis and with aggressiveness in human glioma [24]. Changes in secondary structure of profilin 1 can interfere with the binding of ligands. Profilin 1 itself has only 5 to 15% α-helix [25–27] which, when bound to phosphatidylinositol (4,5) bisphosphate, increases up to 35% [25,26]. A consequence of this change in conformation is the dissociation of actin from the actin—profilin 1 complex [25]. Nitration of Tyr139 [28] and ligand oligomerization [29] both change the affinity of profilin 1 for ligands. Truncation of the C-terminal part is another possible way by which ligand binding can be regulated. The importance of this was observed with bovine profilin 1, which is present in the spleen in two forms, a native one and the other lacking the terminal Gln138 and Tyr139. These residues have been associated, importantly, with the stability of the profilin—actin complex and with the binding of phosphatidylinositol lipids [27].

In our previous study the carboxymonopeptidase cathepsin X was shown to cleave Tyr139 from full-length recombinant profilin 1 [8]. Tyr139 has been confirmed here as a target for cathepsin X carboxypeptidase activity by mutants with changes at the C-terminal end. Of these, Pfn-Tyr139 was chosen on the basis of results obtained on full length recombinant profilin 1. Cathepsin X no longer cleaves after reaching proline at the P2 position [17,30–32]. A second mutant Pfn-Q138P was therefore chosen to prevent any cleavage of profilin 1 at the C-terminal part. The mutant lacking Tyr139 significantly increased migration and invasion of PC-3 cells, suggesting that Tyr139 is the amino acid residue that cathepsin X cleaves under physiological conditions. All the transfected cells express both native and mutated profilin. Pfn-Tyr139 is thus the profilin 1 form after cleavage by cathepsin X. Since there is native profilin 1 still present in transfected PC-3 cells, the effect of total profilin 1 should be changed by adding cathepsin X inhibitor AMS36. Indeed, AMS36 inhibited cathepsin X action on native profilin 1 and decreased the migration of PC-3 cells. In contrast, the addition of recombinant cathepsin X potentiated the effect of total profilin 1 on cell migration. As expected, the results of all tests with Pfn-Tyr139 are in direct contrast to those where AMS36 and specific siRNA were used to study the effects of cathepsin X on profilin 1 function [8].

A proximity ligation assay demonstrated that cathepsin X specific inhibitor AMS36 significantly increased the number of profilin 1—clathrin association signals in PC-3 cells, suggesting that intact profilin 1 binds clathrin. Furthermore, application of AMS36 and siRNA silencing increased the endocytosis of dextran particles by 50% and 25%, respectively. The greater effect of AMS36 can be attributed to partial inhibition of cathepsin B [16], another cysteine protease that can promote endocytosis. To confirm that the endocytosis is clathrin-mediated, chlorpromazine, the inhibitor of clathrin-mediated endocytosis, was applied to neutralize the effect of AMS36 and siRNA. PC-3 cells were tested by fluorescence microscopy for uptake of dextran. Treated cells contained a large number of vesicles of various sizes loaded with dextran particles while, in control cells, vesicles containing dextran particles were small and very sparse. Changes in clathrin-mediated endocytosis were shown also by transferrin conjugated to Alexa Fluor 555. Here the endocytosis increased by 30% in cells with silenced cathepsin X. Clathrin-mediated transferrin uptake was confirmed with the receptor saturation when there was no endocytosis. The role of profilin 1 in endocytosis is not clear. Since it forms complexes with many different ligands, Witke et al. speculated that, in these complexes, cytoskeletal elements, regulatory molecules and other proteins, all essential for endocytosis, are physically brought together [15]. According to this, the actin cytoskeleton could act as a temporary membrane organizer until endocytosis is induced. Profilin 1 was also found to be important for fluid phase endocytosis at higher temperatures in *Saccharomyces cerevisiae* by enhancing ADP/ATP exchange on actin [33] and in *Dictyostelium discoideum*, by regulating actin cytoskeleton polymerization [34,35]. Further, profilin was also found, in *Drosophila*, to be important for phagocytosis [36]. Mouse profilin 2 is known to associate with dynamin 1 and, by that, to interfere with assembly of the endocytic apparatus [37], inhibiting endocytosis of kainate receptor by general regulation of clathrin-mediated endocytosis [38]. Our results confirm the association of human profilin 1 with clathrin-mediated endocytosis by binding clathrin and possibly other ligands at its C-terminal binding site. Clathrin-mediated endocytosis is linked with cancer progression through the regulation of receptor signaling. For example, fibroblast migration was shown to be regulated by endocytosis of platelet-derived growth factor (PDGF) receptor [39], and chemotactic invasion of MDA-MB-231 breast cancer cells and pancreatic ductal adenocarcinoma cells by clathrin-mediated endocytosis of epidermal growth factor (EGF) receptor [40].

The C-terminal part of profilin 1 has been shown here to be important for ligand binding and for its functions related to actin polymerization and cell migration. Further, in prostate tumor cell line PC-3, cleavage of the C-terminal part of profilin 1 by up-regulated cathepsin X could lead to reduced clathrin-mediated endocytosis. The regulation of cathepsin X activity in tumor cells may therefore provide a tool for the development of new strategies for anticancer treatment.

## Supporting Information

S1 Fig Cathepsin X silencing. PC-3 cells were transfected with control or cathepsin X specific siRNA using Lipofectamine. After 48 hours, cell lysates were prepared and the amount of cathepsin X determined with ELISA (**A**) or western blot (B). Mean values of two separate ELISA experiments (in duplicates) and representative image of western blot are shown. *P < 0.05.
(TIF)

S2 Fig Cytotoxicity of chlorpromazine (CHL) towards PC-3 cells. Cells were treated with various concentrations of CHL to determine the working concentration to be used in further experiments. After a 30 minute pre-treatment with CHL, cells were incubated for a further 80 minutes in its presence after adding MTS reagent for cytotoxicity measurement. Each concentration was tested in quadruplicate. **P<0.01.
(TIF)

S1 Table Western blot antibody information. (DOCX)

S2 Table Proximity-ligation assay antibody information. (DOCX)
