# Supplementary figures and images for: Cathepsin X Cleaves Profilin 1 C-Terminal Tyr139 and Influences Clathrin-Mediated Endocytosis
Source: PLoS One. 2015 Sep 1;10(9):e0137217. doi: 10.1371/journal.pone.0137217 (PMC4567178; doi:10.1371/journal.pone.0137217)

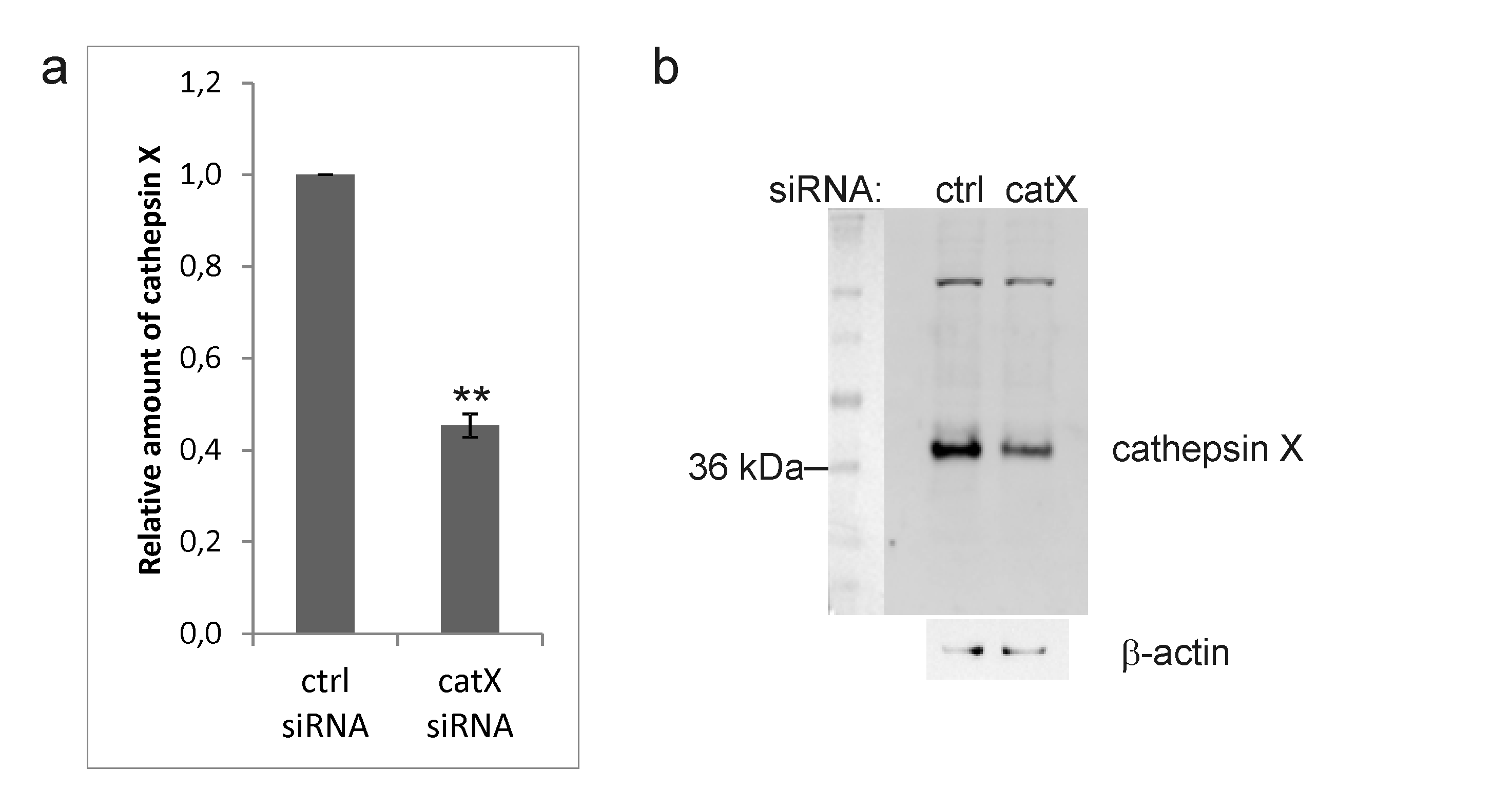

Supplement: S1 Fig — PC-3 cells were transfected with control or cathepsin X specific siRNA using Lipofectamine. After 48 hours, cell lysates were prepared and the amount of cathepsin X determined with ELISA (A) or western blot (B). Mean values of two separate ELISA experiments (in duplicates) and representative image of western blot are shown. *P < 0.05. (TIF) [file pone.0137217.s001.tif]

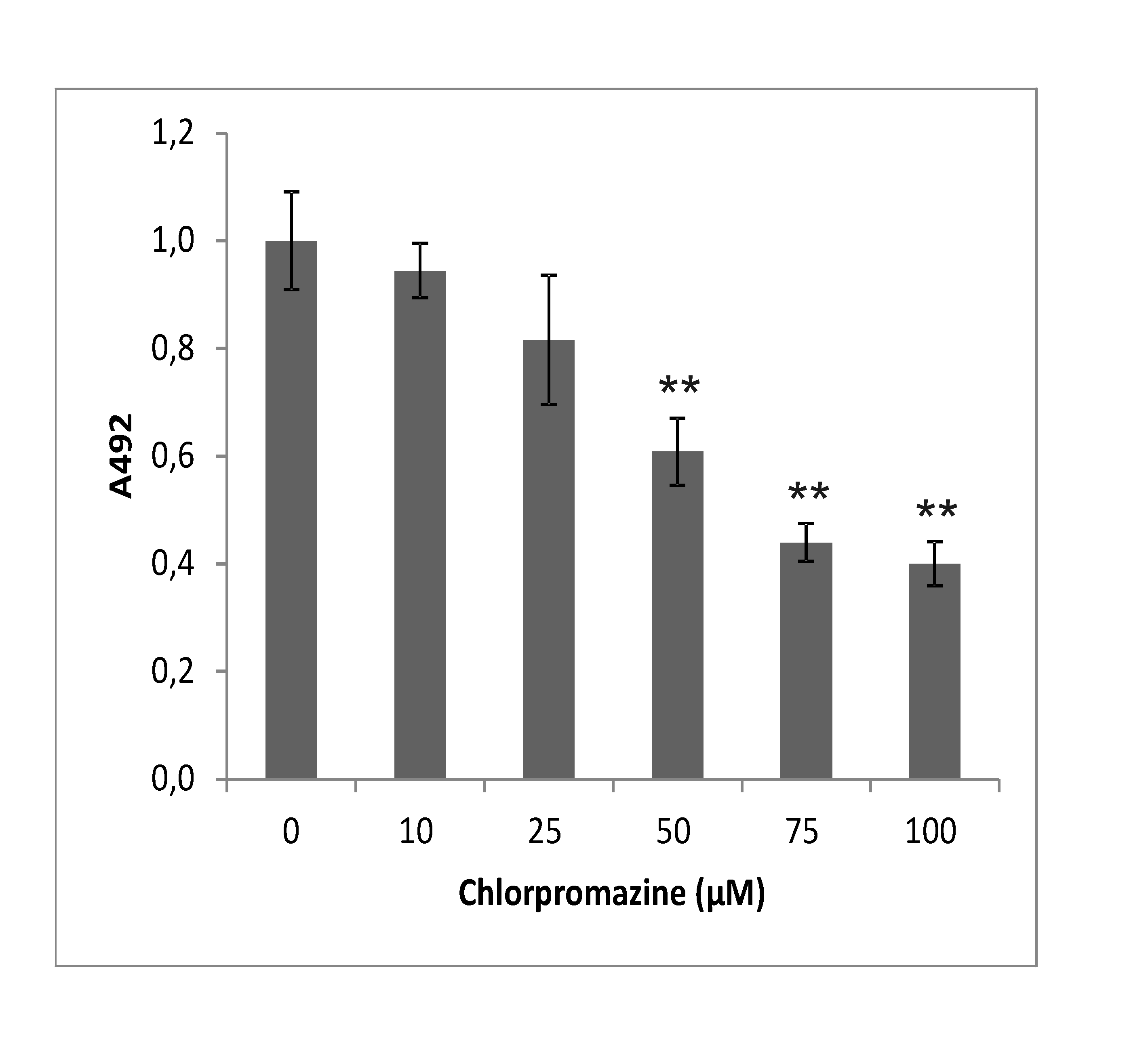

Supplement: S2 Fig — Cells were treated with various concentrations of CHL to determine the working concentration to be used in further experiments. After a 30 minute pre-treatment with CHL, cells were incubated for a further 80 minutes in its presence after adding MTS reagent for cytotoxicity measurement. Each concentration was tested in quadruplicate. **P<0.01. (TIF) [file pone.0137217.s002.tif]
