# Supplementary material for: Cathepsin X Cleaves Profilin 1 C-Terminal Tyr139 and Influences Clathrin-Mediated Endocytosis
Source: PLoS One. 2015 Sep 1;10(9):e0137217. doi: 10.1371/journal.pone.0137217 (PMC4567178; doi:10.1371/journal.pone.0137217)
Supplement: S1 Table — (DOCX) [file pone.0137217.s003.docx]

| Antibody | Supplier (catalogue number) | concentration | dilution |
| --- | --- | --- | --- |
| rabbit anti-profilin 1 pAb | Sigma Aldrich (P7624) | 1 mg/ml | 1:1000 |
| goat anti-rabbit IgG(H+L) HRP conj. | Invitrogen (G21234) | 1 mg/ml | 1:5000 |
| mouse anti-FLAG mAb | Sigma-Aldrich (F1804) | 1 mg/ml | 1:1000 |
| goat anti-mouse IgG/IgM HRP conj. | Milipore (AP130P) | unknown | 1:2500 |
| goat anti-cathepsin X pAb | R&D Systems (AF 934) | 0.2 mg/ml | 1:1000 |
| donkey anti-goat IgG-HRP conj. | Santa Cruz (sc 2020) | 0.2 mg/0.5ml | 1:2500 |
| mouse anti-β-actin mAb | Sigma-Aldrich (A5316) | 1.6 mg/ml | 1:5000 |
| goat anti-mouse IgG/IgM HRP conj. | Milipore (AP130P) | unknown | 1:5000 |

**S1 Table: Western blot antibody information**
