# Supplementary material for: Cathepsin X Cleaves Profilin 1 C-Terminal Tyr139 and Influences Clathrin-Mediated Endocytosis
Source: PLoS One. 2015 Sep 1;10(9):e0137217. doi: 10.1371/journal.pone.0137217 (PMC4567178; doi:10.1371/journal.pone.0137217)
Supplement: S2 Table — (DOCX) [file pone.0137217.s004.docx]

**S2 Table: Proximity-ligation assay antibody information**

| Antibody | Supplier (catalogue number) | concentration | dilution |
| --- | --- | --- | --- |
| mouse anti-clathrin mAb | Abcam (ab2731) | 6 mg/ml | 1:1750 |
| rabbit anti-profilin 1 pAb | Sigma Aldrich (P7624) | 1 mg/ml | 1:200 |
